# 

**Published:** 1905-10-07

**Authors:** 


### NOTICE TO CORRESPONDENTS.

All MSS., Letters, Books for Review, and other matters intended for the Editor should be addressed The Editor, "The Hospital," The Hospital Buildings, 28 & 29 Southampton Street, Strand, W.C.

The Editor cannot undertake to return rejected MS., even when accompanied by stamped directed envelope.

**Notice to Advertisers and Subscribers.**

All Advertisements, Orders for Copies of The Hospital, and Business Communications should be addressed to **THE MANAGER (Not to the Editor),** The Hospital, 28 & 29 Southampton Street, Strand, London, W.C.

The Cost of Subscription, post free, is at follows:—Per week, 3½d.; per quarter, 3s. 3d.; per half-year, 6s. 6d.; per annum, 13s. Foreign and Colonial:—Per annum, 19s. 6d., payable, in all cases, in advance.

**NOTICE.—Vol. XXXVII. of "The Hospital" October 1904 to March 1905), in handsome cloth binding, will shortly be on sale at the office of this Journal, price 10s. 6d. Cases for binding the Numbers contained in this Volume 2s. Index to Vol. XXXVII., 3½d., post free.**

**The Monthly Part for October is now ready. Price 1s., by post, 1s. 3d.**

### Medical Appointments.

**Cardew, Henry J.,** M.R.C.S., L.R.C.P., M.A.Cantab., Senior House Surgeon to the Royal South Hants and Southampton Hospital, Southampton. **Collum, Rowland W.,** L.R.C.P.Lond., M.R.C.S.Eng, Anæsthetist to St. Mary's Hospital, Paddington. **Crinks, V. A.,** L.R.C.P. Lond., M.R.C.S., House Surgeon to the Newport and Monmouthshire Hospital. **Davis, Henry,** M.R.C.S.Eng., Senior Honorary Anæsthetist to St. Mary's Hospital, Paddington. **De Jong, E. M.,** M.R.C.S., L.S.A., Certifying Surgeon under the Factory and Workshop Act for the Lymm District of the County of Chester. **Dishington, T. M.,** M B., Ch.M.Glasg., Outdoor House Surgeon to the Maternity Hospital, Glasgow. **Evans, Richard David,** L.R.C.P.Lond., M.R.C.S.Eng., Medical Officer of Health of the Llandilo (Carmarthenshire) Urban District. **Fisher, Theodore,** M.D.Lond., M.R.C.P Lond., M.R.C.S, Pathologist at the North-Eastern Hospital, London. **Hopkins, C. L.,** M.B., B.C.Cantab., Medical Superintendent of the York City Asylum. **Hutchinson, J. R.,** M.B., Ch.B.Vict., D.P.H.Vict., House Surgeon to the Liverpool Eye and Ear Infirmary. **Knight, Hubert Astley,** M.B., Ch.B.Edin., L.R.C.P. & S.Edin., L.F.P.S.Glasg., D.P.H.Edin. and Glasg., Assistant House Physician at the Bristol Royal Infirmary. **Lucas, Joseph John Scammell,** B.A., M.D.Lond., L.R.C.P., M.R.C.S, Honorary Pathologist to the Bristol Royal Infirmary. **Pairman, James C.,** M.B., Ch.B.Glasg., Indoor House Surgeon to the Maternity Hospital, Glasgow. **Patrick, Howard H.,** M.B., Ch.M.Glasg., Outdoor House Surgeon to the Maternity Hospital, Glasgow **Sheppard, Arthur Lewin,** M.B., B.S.Durh., Junior House Surgeon at the Bristol Royal Infirmary. **Smith, Lionel Singleton,** L.R.C.P.Lond., M.R.C.S., Casualty Officer at the Bristol Royal Infirmary. **Stevenson, Howard,** M.B., B.Ch.R.U.I., F.R.C.S.Irel., House Visiting Surgeon to the Women's Department of the Ulster Hospital, Belfast. **Townroe, Eugene Dunbar,** L.R.C.P.Lond., M.R.C.S., Resident Medical Officer at the Winsley Sanatorium.

### Nurses' Book Page.

### A HANDBOOK FOR NURSES.

By J. K. Watson, M.D., M.B., C.M.

New and Revised Edition. Profusely Illustrated. **5/-** net, **5/4** post free.

### NURSING: Its Theory and Practice.

**Being a complete Text-book of Medical, Surgical, and Monthly Nursing.**

By Percy G. Lewis, M.D., M.R.C.S., L.S.A.

New Edition. Profusely Illustrated.

**3/6** post free.

### SURGICAL WARD-WORK AND NURSING.

By Alexander Miles, M.D.Edin., C.M., F.R.C.S.E.

Second Edition. With particulars and Illustrations of the instruments used in various operations.

**3/6** net, **3/10** post free.

### OPHTHALMIC NURSING.

By Sydney Stephenson, M.B. F.R.C.S.E.

New and Revised Edition.

**3/6** net, **3/10** post free.

### ELEMENTARY ANATOMY AND SURGERY FOR NURSES.

By William McAdam Eccles, M.S.Lond., M.B., F.R.C.S., L.R.C.P.

**2/6** post free.

### ELEMENTARY PHYSIOLOGY FOR NURSES.

By C. F. Marshall, M.D., B.Sc., F.R.C.S.

A valuable help to Physiology for Probationers. Crown 8vo. Illustrated, cloth.

**2/-** post free.

### NURSING IN DISEASES OF THE THROAT, NOSE, AND EAR.

By P. MacLeod Yearsley, F.R.C.S.Eng., M.R.C.S., L.R.C.P.Lond.

**2/6** post free.

### HOSPITAL SISTERS AND THEIR DUTIES.

By Eva C. E. Lückes, Matron, London Hospital. Third Edition.

**2/6** post free.

### A COMPLETE HANDBOOK OF MIDWIFERY.

**The Best Text-Book for the C.M.B.**

By J. K. Watson, M.D.Edin.

143 Illustrations. **6/-** net, **6/4** post free.

### A PRACTICAL HANDBOOK OF MIDWIFERY.

By Francis W. Haultain, M.D.

Illustrated. **6/-** net, **6/3** post free.

### PRACTICAL GUIDE TO SURGICAL BANDAGING AND DRESSINGS.

By Wm. Johnson Smith, F.R.C.S.

A handy and useful reference book. Suitable size for apron pocket.

**2/-** post free.

### THE EXAMINATION OF THE URINE.

**Compiled for the Use of Nurses.**

By J. K. Watson, M.D., M.B., C.M.

**1/-** net, **1/1** post free.

### THE NURSES' PRONOUNCING DICTIONARY OF MEDICAL TERMS AND NURSING TREATMENT.

By Honnor Mortrn.

New Edition, with phonetic pronunciations. Edited by Mary I. Burdett.

**2/-** post free.

### NURSING HINTS TO PROBATIONERS ON PRACTICAL WORK.

By Annesley Voysey.

Cloth, Illustrated, **2/-** net, **2/4** post free.

This work fills a distinct hiatus in Nursing literature. It will prove of great assistance to the Nurse in the early stages of her career. [*Ready next week.*

### MEDICAL ELECTRICITY AND THE LIGHT TREATMENT.

By Kate Neale, Sister in Charge Light Department, Guy's Hospital.

Cloth, profusely Illustrated, **2/6** net, **2/9** post free.

A practical book on a subject hitherto untouched from a Nursing standpoint. [*Ready next week.*

### ART OF MASSAGE.

By Mrs. Creighton Hale.

Second Edition. **6/-** post free.

---

*Publishers of Nursing Text-books, Charts, Case-books, &c., of all Descriptions.*

SEND FOR NEW CATALOGUE OF NURSING MANUALS.

**THE SCIENTIFIC PRESS, Ltd.,**

**28 and 29 Southampton Street, Strand, London, W.C.**

